# Use of the GRADE approach in health policymaking and evaluation: a scoping review of nutrition and physical activity policies

**DOI:** 10.1186/s13012-020-00984-2

**Published:** 2020-05-24

**Authors:** Jasmin Zähringer, Lukas Schwingshackl, Ani Movsisyan, Jan M. Stratil, Sara Capacci, Jürgen M. Steinacker, Sarah Forberger, Wolfgang Ahrens, Daniela Küllenberg de Gaudry, Holger J. Schünemann, Joerg J. Meerpohl, On behalf of the PEN consortium

**Affiliations:** 1 Institute for Evidence in Medicine, Medical Center - University of Freiburg, Faculty of Medicine, University of Freiburg, Freiburg, Germany; 2 Institute for Medical Information Processing, Biometry and Epidemiology (IBE), Pettenkofer School of Public Health, LMU Munich, Munich, Germany; 3 Department of Statistical Sciences, University of Bologna, Bologna, Italy; 4 Division of Sports- and Rehabilitation Medicine, Medical Center, Ulm University Hospital, Ulm, Germany; 5 Department Prevention and Evaluation, Leibniz-Institute for Prevention Research and Epidemiology – BIPS, Bremen, Germany; 6 McMaster GRADE Centre and Department of Health Research Methods, Evidence, and Impact, McMaster University Health Sciences Centre, Hamilton, Ontario Canada; 7 Cochrane Germany, Cochrane Germany Foundation, Freiburg, Germany

**Keywords:** Policy evaluation, GRADE, Evidence-based, Nutrition, Physical activity, Health policymaking, Policy Evaluation Network

## Abstract

**Background:**

Nutrition and physical activity policies have the potential to influence lifestyle patterns and reduce the burden of non-communicable diseases. In the world of health-related guidelines, GRADE (Grading of Recommendations, Assessment, Development and Evaluation) is the most widely used approach for assessing the certainty of evidence and determining the strength of recommendations. Thus, it is relevant to explore its usefulness also in the process of nutrition and physical activity policymaking and evaluation.

The purpose of this scoping review was (i) to generate an exemplary overview of documents using the GRADE approach in the process of nutrition and physical activity policymaking and evaluation, (ii) to find out how the GRADE approach has been applied, and (iii) to explore which facilitators of and barriers to the use of GRADE have been described on the basis of the identified documents. The overarching aim of this work is to work towards improving the process of evidence-informed policymaking in the areas of dietary behavior, physical activity, and sedentary behavior.

**Methods:**

A scoping review was conducted according to current reporting standards. MEDLINE via Ovid, the Cochrane Library, and Web of Science were systematically searched up until 4 July 2019. Documents describing a body of evidence which was assessed for the development or evaluation of a policy, including documents labeled as “guidelines,” or systematic reviews used to inform policymaking were included.

**Results:**

Thirty-six documents were included. Overall, 313 GRADE certainty of evidence ratings were identified in systematic reviews and guidelines; the strength of recommendations/policies was assessed in four documents, and six documents mentioned facilitators or barriers for the use of GRADE. The major reported barrier was the initial low starting level of a body of evidence from non-randomized studies when assessing the certainty of evidence.

**Conclusion:**

This scoping review found that the GRADE approach has been used for policy evaluations, in the evaluation of the effectiveness of policy-relevant interventions (policymaking), as well as in the development of guidelines intended to guide policymaking. Several areas for future research were identified to explore the use of GRADE in health policymaking and evaluation.

Contributions to the literature

- Policies in the area of nutrition and physical activity should be based on the best available scientific evidence in order to have the best possible impact.
- The GRADE approach is a tool to form trustworthy evidence-informed recommendations; thus, it may contribute to improving the process of evidence-informed policymaking. It is already being used in policy evaluation, in evaluations of effectiveness, and in the development of guidelines.
- This research did not reveal actual policies or formulation processes from governmental bodies so future research should focus on assessing the views of policymakers on the usefulness of GRADE to inform their decisions.

## Background

According to the most recent report by the Global Burden of Disease study group, non-communicable diseases (NCDs), such as cardiovascular disease, cancer, and type 2 diabetes, accounted for 64.8% of total deaths worldwide in 2017 [1]. Major risk factors for the development of NCDs are obesity, dyslipidemia, insulin resistance, and hypertension [1–3]. Suboptimal dietary behavior and physical inactivity, including increased sedentary behavior, are related to these risk factors. Therefore, adherence to an optimal diet and physical activity recommendations can contribute to the prevention of obesity and several NCDs [4].

On a public health level, policies are important instruments that can be used to change people’s behavior [5]. While the definition of what constitutes a policy remains a topic of debate, for the purpose of this review, we use the term “policy” both for (a) interventions implemented at a population level, as well as for (b) recommendations on a specific course of action to be implemented by a government or other public body (see Table 1).

**Table 1:** Overview of the definitions “policy” and examples for policymaking in the present scoping review

**Definition of policy in this scoping review:** • Decisions, plans, and actions that are undertaken to achieve specific health care goals within a society (World Health Organization and Policy Evaluation Network) [6, 7]. • A policy can be seen as a macro-level intervention or is followed by an intervention. • A policy is supposed to change a system not an individual (addresses group of people) [8]. A policy that has a positive effect on one issue can have a negative effect on another issue at the same time. **Distinctions made for identified document types:** • Policy evaluation: evaluation of macro-level laws/regulations, adopted and implemented at the level of geographically defined political or administrative units, such as supra-national organizations, states, regions, or municipalities. • Evidence of effectiveness for policymaking: assessment of interventions or evidence on information which has the potential to become a policy intervention. • Recommendations to guide policymaking: guidelines and recommendations by institutions to adopt or implement interventions which have the potential to become a policy.	

Policies have the potential to influence lifestyle patterns within a population [5]. Certain good-practice characteristics of successful policies on optimal diets and physical activity have been identified in recent research, such as the use of the theory of change, analyses of target populations and target behavior, content development and management, multidimensionality, and appropriate consideration of practitioners and settings [9]. For example, interventions such as mass media campaigns, school programs, or changes of the environment through communities showed to be effective in increasing physical activity levels among a population, especially when they operate on multiple levels and contain multiple components [10]. However, to be maximally effective, they require regular evaluation and improvement. The Policy Evaluation Network (PEN), a consortium funded by the European Union, was founded to help improve policymaking in the areas of dietary behavior, physical activity, and sedentary behavior by evaluating existing policies [6, 11]. This scoping review is part of the PEN-project, with a special focus on policies that explicitly target, or are likely to impact upon, population health, in particular through NCDs.

To increase the acceptance and impact of polices, it might be helpful if they adhered to principles widely accepted, for instance, similar to health guidelines, i.e., they should be transparent, structured, and evidence-informed [12, 13]. Within the PEN-project, it is explored if these guideline standards, which also include the assessment of the certainty of evidence, should be considered when developing and evaluating a policy [14].

The Grading of Recommendations, Assessment, Development and Evaluation (GRADE) approach and the GRADE Evidence to Decision (EtD) frameworks are important methods for the development and evaluation of recommendations for practice and can therefore also help to make evidence-informed decisions on a population level [15–17]. GRADE is a well-recognized tool to facilitate structured decision-making and is potentially the most widely used approach for assessing the certainty of evidence and determining the strength of a recommendation [18]. It is standard practice to use the GRADE approach to form trustworthy evidence-informed recommendations, especially in the development of guidelines in the broader field of medicine. For this reason, it seems sensible to explore its potential in the process of developing and evaluating policies concerned with nutrition and physical activity [16].

The primary aim of this scoping review is to investigate how and to what degree the GRADE approach has contributed to policy development in the context of nutrition and physical activity. To this end, the following steps were taken: (i) an overview of documents using the GRADE approach in the context of nutrition and physical activity policymaking and evaluation was compiled, (ii) methods of applying the GRADE approach (e.g., by rating the certainty of evidence or the strength of a recommendation) were recorded, and (iii) facilitators of and barriers to the use of GRADE described in the identified documents were identified and explored.

## Methods

This scoping review was conducted according to the methodology of the Joanna Briggs Institute’s (JBI) Reviewers’ Manual 2015 and is reported following the PRISMA (Preferred Reporting Items for Systematic reviews and Meta-Analyses) extension for scoping reviews, the PRISMA-ScR Statement (Additional file 1) [19, 20]. To complement our scoping review, a survey was conducted by contacting European health policymaking institutions to further investigate the use of GRADE in nutrition and physical activity policymaking and evaluation and to potentially identify additional policy documents.

### Search strategy

First, policy evaluations in the form of systematic reviews and other relevant documents published in peer-reviewed journals were identified through searches in the databases MEDLINE, Web of Science, and the Cochrane Library. The search strategy was developed with the support of an information specialist and adapted for each database accordingly. Databases were searched up until 4 of July 2019, and the search was not restricted to any language. The MEDLINE (Ovid) search strategy is presented in Additional file 2. Moreover, forward citation tracking and reference checking from included documents were performed.

Secondly, an internet-based search through the Google™ search engine and through Google Scholar™ was performed to identify gray literature and different relevant institutions and organizations, such as the Food and Agriculture Organization for the United Nations (FAO), World Health Organization (WHO), World Cancer Research Fund International (WCRF), the National Institute for Health and Care Excellence (NICE), and the Campbell Collaboration. Following this, the types of documents relevant for policymaking and which address dietary behavior, physical activity, and/or sedentary behavior, such as guidelines, reports, or systematic reviews, were searched on the websites of relevant organizations. Documents were found both through the Google search directly and also retrieved from the websites of those organizations and institutions identified in the search.

Thirdly, an extensive overview of existing policies including policy evaluations from the NOURISHING-database by the WCRF was considered [21]. Lastly, science platforms and databases such as ProQuest, Dimensions, BASE, Metager, Oaister, and the Political Science Database (Berkeley) were searched.

### Inclusion criteria

Because of the lack of a standard definition of the term “policy” across various areas of health policy and policy decision-making, the current search also aimed to identify documents that might not have used the term “policies” or “policy evaluations.” We focused on documents that described a body of evidence which was assessed for the development or evaluation of a policy, including documents labeled as “guidelines,” or systematic reviews used to inform policymaking [22]. Furthermore, we established criteria for interventions and/or recommendations (i.e., policies) to be eligible. Documents had to meet the following criteria to be included in this scoping review:

(i) At least one intervention or recommendation of a policy document targeted the PEN-related outcomes: dietary behavior, physical activity, or sedentary behavior.
(ii) Recommendations targeted a population rather than individuals. This means that at least one intervention or recommendation had to fit in one of the following categories: school program (only if mandatory to school children, e.g., more physical exercise lessons or change in school meal offerings), mass media (e.g., commercials, poster campaigns), infrastructure (e.g., standing desks), financial incentive (e.g., taxation on sugar or fat), change of offerings (e.g., portion/package sizes), nutrition labeling, and population education (in the sense of recommendations, e.g., guidelines). These categories were established by reviewing a set of pre-identified documents.
(iii) Specific measures or action plans (potential policies) that were or could be implemented by a government or an inter-governmental organization such as the WHO (e.g., labeling and school programs).
(iv) In addition to meeting criteria i–iii, GRADE was used to assess the certainty of evidence or the strength of recommendations or reasons for not using GRADE were explicitly noted.

### Exclusion criteria

There were three exclusion criteria:

(i) Since the GRADE approach is primarily applicable in the context of evidence syntheses within systematic reviews, health technology assessments (with or without meta-analyses) or guidelines, surveys, and primary studies (e.g., randomized controlled trials (RCTs) or non-randomized studies (NRSs)) were excluded.
(ii) Documents concerning the methodological aspects of policy implementation were excluded.
(iii) Documents about interventions that specifically target individuals instead of a population were also excluded.

### Selection process of sources of evidence

First, title and abstract screening was performed by one reviewer (JZ). Only clearly irrelevant references were excluded at this stage. Second, for all potentially relevant references, full-text publications were obtained and checked for final inclusion by two reviewers (JZ, LS) independently. Uncertainties were resolved through discussion with a third author (JJM).

### Data extraction

For included documents, one reviewer (JZ) extracted the following characteristics: name of first author, year of publication, document type, study aim, outcome of interventions (physical activity, dietary behavior, sedentary behavior, multi-component), design of primary studies included in the document, policy/intervention(s), target group/study setting, use of GRADE, GRADE ratings for relevant outcomes (including down- and upgrading factors), facilitators, and barriers to apply GRADE. The data extraction form was piloted and proved to be applicable to all types of retrieved documents. Data extraction was verified by a second reviewer (LS).

### Data categorization and presentation

The documents were categorized according to policy evaluation, evidence of effectiveness for policymaking, or a recommendation to guide policymaking (see Table 1). Furthermore, we categorized the documents according to the applied interventions (type of policy) in the studies (e.g., school program and mass media), according to the setting of the studies (e.g., workplace and community) and according to PEN-relevant outcomes (dietary behavior, physical activity, and sedentary behavior). All these characteristics were collected for each document and were displayed in Table 2. In further tables, we displayed the GRADE ratings, facilitators, and barriers for the use of GRADE (Table 3), as well as the strengths of recommendations (Additional file 4).

**Table 2:** Characteristics of included studies

Author	Document type	Aim	Policy (intervention)	Setting
Al-Khudairy et al. [23]	Evidence of effectiveness for policymaking (non-governmental)	To assess the effects of diet, physical activity and behavioral interventions for the treatment of overweight or obese adolescents aged 12 to 17 years.	School program	Schools, population level
Baker et al. [24]	Evidence of effectiveness for policymaking (non-governmental)	To assess the effects of community-wide, multi-strategic interventions upon community levels of physical activity.	Mass media, school programs, infrastructure	Schools, communities
Balogun et al. [25]	Evidence of effectiveness for policymaking (non-governmental)	To examine interventions which aim to encourage women to breastfeed.	Mass media	Population level
Carducci et al. [26]	Evidence of effectiveness for policymaking (non-governmental)	To understand the impact of food environment interventions on diet-related health outcomes in school-age children and adolescents.	Nutrition labeling, change in offerings, school program, mass media, infrastructure	Schools, workplace, communities, population level
Crockett et al. [27]	Policy evaluation (non-governmental)	To examine whether nutritional labels (i.e., labels providing information about nutritional content) persuade people to buy or consume different (healthy) kinds of food.	Nutrition labeling	Population level
Cushing et al. [28]	Evidence of effectiveness for policymaking (non-governmental)	To evaluate the overall effectiveness of health promotion interventions in children and adolescents.	Portion size, change in offerings, school program	Communities, schools, population level
Dobbins et al. [29]	Evidence of effectiveness for policymaking (non-governmental)	To summarize the evidence of the effectiveness of school-based interventions in promoting physical activity and fitness in children and adolescents.	School program, infrastructure	Schools
Dyson et al. [30]	Recommendations to guide policymaking (non-governmental)	To present the latest evidence-based nutrition guidelines for the prevention and management of diabetes.	Population education	Population level
Elvsaas et al. [31]	Evidence of effectiveness for policymaking (non-governmental)	To assess the effect of multicomponent lifestyle interventions including two or more lifestyle components on change in BMI in children and adolescents.	School program, mass media infrastructure	Schools, population level
Erickson et al. [32]	Evidence of effectiveness for policymaking (non-governmental)	To systematically review guidelines on sugar intake and assess consistency of recommendations, methodological quality of guidelines, and the quality of evidence supporting each recommendation.	Population education	Population level
Flatz et al. [33]	Evidence of effectiveness for policymaking (non-governmental)	To determine the effectiveness of interventions implemented through sporting organizations to promote physical activity, healthy diet, reductions in alcohol consumption or tobacco use.	Mass media	Population level
Freak-Poli et al. [34]	Evidence of effectiveness for policymaking (non-governmental)	To assess the effectiveness of pedometer interventions in the workplace for increasing physical activity and improving subsequent health outcomes.	Financial incentive, infrastructure	Workplace
Heise et al. [35]	Policy evaluation (governmental)	To assess the effects of taxation of SSBs, on SSB consumption, energy intake, overweight, obesity, and other adverse health outcomes in the general population.	Financial incentive	Population level
Heise et al. [36]	Evidence of effectiveness for policymaking (non-governmental)	To assess the effectiveness of voluntary participation in community gardening compared on overweight/obesity and associated health outcomes.	School program, infrastructure	Population level, Schools
Hodder et al. [37]	Evidence of effectiveness for policymaking (non-governmental)	To assess the effectiveness, cost-effectiveness, and associated adverse events of interventions designed to increase the consumption of fruit, vegetables, or both among children aged 5 years and under.	Change of offerings, school program, infrastructure	Population level, schools
Hollands et al. [38]	Evidence of effectiveness for policymaking (non-governmental)	To assess the effects of interventions involving exposure to different sizes or sets of physical dimensions of a portion, package, individual unit, or item of tableware on unregulated selection or consumption of food, alcohol, or tobacco products in adults and children.	Portion/package size	Population level
Langford et al. [39]	Policy evaluation (non-governmental)	To assess the effectiveness of the WHO Health Promoting Schools (HPS) framework in improving the health and well-being of students and their academic achievement.	WHO program: Health Promoting Schools: School program, population education, infrastructure, change in offerings	Schools, population level
Lhachimi et al. [40]	Policy evaluation (governmental)	To assess the effects of taxation of fat content in food on consumption of total fat and saturated fat, energy intake, overweight, obesity, and other adverse health outcomes in the general population.	Financial incentives	Population level
Martin et al. [41]	Evidence of effectiveness for policymaking (non-governmental)	To evaluate the effect of interventions which included an SB outcome measure in adults.	Financial incentives, infrastructure	Workplace, population level
Matwiejczyk et al. [42]	Evidence of effectiveness for policymaking (non-governmental)	To examine (1) the effectiveness of interventions to promote healthy eating in children aged 2–5 years attending center-based childcare; (2) intervention characteristics which are associated with successfully promoting healthy eating in pre-schoolers; and (3) recommendations for child-health directed policies and practices.	School program, change in offerings, population education	Schools
McLaren et al. [43]	Policy evaluation (governmental)	To assess the impact of population-level interventions for dietary sodium reduction in government jurisdictions worldwide and to assess the differential impact of those initiatives by social and economic indicators.	Change in offerings, Nutrition labeling	Population level
Mosdol et al. [44]	Evidence of effectiveness for policymaking (non-governmental)	To determine the effect of mass media interventions targeting adult, ethnic minorities with messages about physical activity, dietary patterns, tobacco use, or alcohol consumption to reduce risk of NCDs.	Mass media	Population level
NICE [45]	Recommendations to guide policymaking (non-governmental)	To improve the physical environment to encourage and support physical activity. The aim is to increase the general population’s physical activity levels.	Population education	Population level
Oakman et al. [46]	Evidence of effectiveness for policymaking (non-governmental)	To assess systematically the available evidence on the effectiveness of work-based interventions on the work ability of employees.	(Financial) incentives, infrastructure	Workplace
Okely et al. [47]	Recommendations to guide policymaking (non-governmental)	To outline the process and outcomes for adapting the Canadian 24-Hour Movement Guidelines for the Early Years to develop the Australian 24-Hour Movement Guidelines for the Early Years guided by the GRADE-ADOLOPMENT framework.	Population education	Population level
Pfinder et al. [48]	Policy evaluation (governmental)	To assess the effects of taxation of unprocessed sugar or sugar-added foods in the general population on the following: 1. Consumption of unprocessed sugar or sugar-added foods; 2. Prevalence and incidence of overweight and obesity; and 3. Prevalence and incidence of diet-related health conditions.	Financial incentive	Population level
von Philipsborn et al. [49]	Policy evaluations (governmental)	To assess the effects of environmental interventions (excluding taxation) targeted at sugar-sweetened beverages or low-calorie alternatives to sugar-sweetened beverages on consumption levels, diet-related anthropometric measures, and health outcomes, and on any reported unintended consequences or adverse outcomes.	Nutrition labeling	Population level
Salam et al. [50]	Evidence of effectiveness for policymaking (non-governmental)	To assess the impact of lifestyle interventions (including dietary interventions, physical activity, behavioral therapy or any combination of these interventions) along with the contextual factors to prevent and manage childhood and adolescent obesity.	Change in offerings, school program	Schools, communities, population level
Shrestha et al. [51]	Evidence of effectiveness for policymaking (non-governmental)	To evaluate the effectiveness of workplace interventions to reduce sitting at work.	Infrastructure, population education	Workplace
Tremblay et al. [52]	Recommendations to guide policymaking (non-governmental)	To outline the process and outcomes for the development of the first Canadian Physical Activity Guidelines for the Early Years (aged 0–4 years) and to provide a summary of this process and present the guidelines themselves.	Population education	Population level
Tremblay et al. [53]	Recommendations to guide policymaking (non-governmental)	To outline the process and outcomes for the development of the Canadian 24-Hour Movement Guidelines for Children and Youth: An Integration of Physical Activity, Sedentary Behavior, and Sleep.	Population education	Population level
Verweij et al. [54]	Evidence of effectiveness for policymaking (non-governmental)	To critically examine the effectiveness of workplace interventions targeting physical activity, dietary behavior, or both on weight outcomes.	Population education	Workplace
WHO [55]	Recommendations to guide policymaking (inter-governmental)	To provide guidance on appropriate assessment and management of infants and children at primary health-care facilities, in order to reduce the risk of overweight and obesity among children, including those living in settings where both undernutrition and overweight/obesity are prevalent.	Population education	Population level
WHO [56]	Recommendations to guide policymaking (inter-governmental)	To provide recommendations on the consumption of potassium to reduce NCDs in adults and children.	Population education	Population level
WHO [57]	Recommendations to guide policymaking (inter-governmental)	To provide recommendations on the consumption of sodium to reduce NCDs in most adults and children.	Population education	Population level
WHO [58]	Recommendations to guide policymaking (inter-governmental)	To provide recommendations on the intake of free sugars to reduce the risk of NCDs in adults and children, with a particular focus on the prevention and control of unhealthy weight gain and dental caries.	Population education	Population level

*SSB* sugar-sweetened beverages, *SB* sedentary behavior, *NCD* non-communicable disease, *GRADE-ADOLOPMENT* GRADE Evidence to Decision frameworks for adoption, adaptation, and de novo development of trustworthy recommendations

**Table 3:** Studies that applied the GRADE approach for rating PEN-relevant outcomes

Author	GRADE rating	Outcome	Reason for downgrading
Al-Khudairy et al. [23]	⊕⊕◯◯ Low	Change in BMI	Inconsistency, indirectness
	⊕⊕◯◯ Low	Adverse events	RoB, limited information
	⊕⊕◯◯ Low	Health-related quality of life	RoB, inconsistency
Baker et al. [24]	⊕⊕◯◯ Low	Physical activity in % (end of intervention to 6 years) and energy expenditure	Inconsistency, imprecision
	⊕⊕⊕⊕ High	Physical activity in % (end of intervention to 3 years, 4 months)	
	⊕⊕⊕◯ Moderate	Physical activity, average daily minutes of moderate to vigorous (24 months)	Findings based on a single study
Balogun et al. [25]	⊕⊕◯◯ Low	Initiation of breastfeeding	Inconsistency and RoB
	⊕◯◯◯ Very Low	Early initiation of breastfeeding	Inconsistency, RoB, wide CI
Crockett et al. [27]	⊕◯◯◯ Very low	Food purchased from vending machines	Very serious RoB (2 levels), imprecision
	⊕◯◯◯ Very low	Food purchased from a grocery store	NRSs, RoB, indirectness
	⊕◯◯◯ Very low	Potential harms (high-energy snack foods consumed with misleading low fat/energy labels in laboratory settings)	RoB, Inconsistency, indirectness
	⊕⊕◯◯ Low	Food purchased in restaurants (labels on menus)	Very serious RoB (2 levels)
	⊕⊕◯◯ Low	Food consumed in laboratory settings (labels on menus or labels placed on a range of food options)	Imprecision, indirectness
	⊕⊕◯◯ Low	Food consumed in laboratory settings (single snack food or drink option)	RoB, indirectness
Cushing et al. [28]	⊕⊕⊕◯ Moderate	Overall assessment, diet, physical activity, and smoking	Inconsistency or RoB
Dobbins et al. [29]	⊕⊕◯◯ Low	Television viewing, physical activity rates, physical activity duration, mean systolic/diastolic blood pressure, BMI	Inconsistency, imprecision (same reasons for each outcome)
Elvsaas et al. [31]	⊕⊕⊕◯ Moderate	BMI 6 months, BMI 12 months, BMI *Z* score 6 months and BMI *Z* score 12 months	Inconsistency (same reason for each outcome)
	⊕⊕◯◯ Low	BMI 24 months	Inconsistency, imprecision
	⊕⊕⊕⊕ High	BMI *Z* score 24 months	
Freak-Poli et al. [34]*		Workplace pedometer programs vs. alternative physical activity program:	
	⊕⊕◯◯ Low	Physical activity	RoB, imprecision
	⊕⊕◯◯ Low	BMI	RoB, imprecision
	⊕⊕◯◯ Low	Systolic blood pressure	RoB, imprecision
	⊕⊕◯◯ Low	LDL cholesterol	RoB, imprecision
		Workplace pedometer programs compared to no intervention:	
	⊕◯◯◯ Very low	Physical activity	NRSs, RoB
	⊕◯◯◯ Very low	BMI	RoB, imprecision, inconsistency
	⊕⊕◯◯ Low	Systolic blood pressure	RoB, imprecision, inconsistency
Hodder et al. [37]		For all intervention types:	
	⊕◯◯◯ Very low	Short-term impact (< 12 months) child vegetable intake	Inconsistency, RoB, imprecision
	⊕◯◯◯ Very low	Short-term impact on cost-effectiveness and unintended adverse events	RoB, imprecision, publication bias (same reasons for each outcome)
		Intervention: child nutrition education	
	⊕⊕◯◯ Low	Short-term impact child vegetable intake	RoB, imprecision
Hollands et al. [38]	⊕⊕⊕◯ Moderate	Consumption (in general, among adults and among children), selection without purchase (in general and among adults)	RoB (same reason for each outcome)
	⊕⊕◯◯ Low	Selection without purchase among children	RoB, imprecision
Langford et al. [39]	⊕⊕⊕◯ Moderate	Obesity or overweight or body size	Inconsistency
	⊕⊕◯◯ Low	Nutrition	Inconsistency, RoB
	⊕⊕⊕◯ Moderate	Body image or eating disorder	RoB
	⊕⊕◯◯ Low	Physical activity, alcohol, substance use, sexual health	Inconsistency, RoB (same reasons for each outcome)
Martin et al. [41]	⊕⊕⊕◯ Moderate	Effect of lifestyle interventions	RoB
	⊕⊕⊕◯ Moderate	Effect of physical activity/sedentary behavior interventions	RoB
	⊕⊕⊕◯ Moderate	Effect of physical activity interventions	RoB
	⊕⊕◯◯ Low	Effect of sedentary behavior interventions	Imprecision, RoB
McLaren et al. [43]	⊕◯◯◯ Very low	Salt intake in grams per day (overall, men and women)	NRSs, inconsistency, RoB
NICE (physical activity) [45]*^,^**	⊕⊕◯◯ Low	Total physical activity as measured by total time spent in physical activity	NRSs**, RoB, imprecision
	⊕⊕◯◯ Low	Total sedentary time as measured by the total time spent sitting	NRSs**, RoB, imprecision
	⊕◯◯◯ Very low	Changes to transport as measured by % of car drivers switching to public transport	NRSs**, RoB (2×), imprecision
	⊕◯◯◯ Very low	Active travel as measured by the average time spent in active commuting	NRSs**, RoB (2×), imprecision
	⊕◯◯◯ Very low	Physical activity in everyday life as measured by the average time spent in recreational walking and cycling	NRSs**, RoB (2×), imprecision
	⊕⊕◯◯ Low	Changes to transport as measured by changes in proportion of journeys to work made by active travel (proximity)	NRSs**, RoB, indirectness
	⊕◯◯◯ Very low	Public transport use (as a proxy of physical activity) as measured by bus use	NRSs**, RoB (2×), imprecision
Oakman et al. [46]	⊕⊕⊕◯ Moderate	Effect of individually focused workplace interventions on work ability	RoB
	⊕◯◯◯ Very low	Effect of multilevel focused workplace interventions on work ability	RoB (2 levels), imprecision
Shrestha et al. [51]*			
	⊕⊕◯◯ Low	Sit-stand desks without information	RoB, imprecision
	⊕◯◯◯ Very low	Treadmill desk with counseling	Imprecision, RoB (2 levels)
	⊕⊕◯◯ Low	Workplace policy changes (walking strategies)	RoB, imprecision
	⊕⊕◯◯ Low	Workplace policy changes (short vs. long break)	Imprecision, RoB
	⊕⊕◯◯ Low	Information, feedback, and counseling	Imprecision, RoB
	⊕⊕◯◯ Low	Prompts combined with information	Imprecision, RoB
	⊕◯◯◯ Very low	Multi-component intervention	Imprecision, RoB, inconsistency
Verweij et al. [54]*	⊕⊕⊕◯ Moderate	Bodyweight (physical activity and diet, follow-up 6–18 months)	Inconsistency
	⊕⊕◯◯ Low	Bodyweight (phyical activity, follow-up 2––12 months)	RoB, imprecision
	⊕⊕⊕◯ Moderate	BMI (physical activity and diet, follow-up 6–18 months)	RoB
	⊕⊕◯◯ Low	BMI (physical activity, follow-up 2–12 months)	RoB imprecision
	⊕⊕⊕◯ Moderate	Body fat (physical activity and diet, follow-up 6-9 months)	Imprecision
	⊕⊕◯◯ Low	Waist circumference (physical activity and diet, follow-up 24 weeks to 1 year)	Inconsistency, imprecision
	⊕◯◯◯ Very low	Waist–hip ratio (cm) (physical activity and diet; follow-up 3–18 months)	Only one study available
von Philipsborn et al. [49]*	⊕⊕⊕◯ Moderate	Traffic-light labeling on SSB sales	NRSs, upgraded for magnitude of effect
	⊕◯◯◯ Very low	Improved access to drinking water in schools on SSB intake	RoB, NRSs, imprecision
	⊕⊕⊕◯ Moderate	Price-increase on SSB sales	NRSs, upgraded for dose-response gradient and magnitude of effect
	⊕◯◯◯ Very low	Voluntary food and beverage industry initiatives to improve the nutritional quality of the whole food supply on SSB sales	NRSs, RoB
	⊕⊕◯◯ Low	Healthier default beverages in children’s menus in restaurants on SSB sales	NRS, upgraded for magnitude of effect, downgraded for RoB
	⊕⊕⊕◯ Moderate	Government food benefit programs with incentives for buying fruit and vegetables and restrictions on the purchase of SSB	RoB
	⊕⊕⊕◯ Moderate	Improved access to low-calorie beverages in the home environment on SSB intake	RoB
WHO (potassium) [56]*			
	⊕◯◯◯ Very low	Cardiovascular disease	NRSs, imprecision
	⊕⊕◯◯ Low	Stroke	NRSs
	⊕◯◯◯ Very low	Coronary heart disease	NRSs, imprecision
	⊕◯◯◯ Very low	All-cause mortality	Only one study, imprecision
	⊕⊕⊕⊕ High	Resting systolic blood pressure	
	⊕⊕⊕⊕ High	Total cholesterol	
	⊕⊕⊕⊕ High	Plasma noradrenaline	
WHO (primary health-care) [55]	⊕◯◯◯ Very low	BMI with dietary intervention (children aged 0–18 years)	NRSs, indirectness
	⊕◯◯◯ Very low	BMI with dietary and/or physical activity interventions (children aged 0–5 years)	NRSs, indirectness
	⊕◯◯◯ Very low	BMI with physical activity interventions (children aged 0–18 years)	NRSs, indirectness
	⊕◯◯◯ Very low	BMI with physical activity interventions (children aged 0–5 years)	NRSs, indirectness
	⊕⊕◯◯ Low	BMI with specialist setting for treatment	Very serious indirectness
WHO (sodium) [57]*			
	⊕◯◯◯ Very low	Cardiovascular disease (indicates increased risk with increased sodium intake)	NRSs, imprecision
	⊕⊕⊕◯ Moderate	Cardiovascular disease (indicates decreased risk with decreased sodium intake)	Imprecision
	⊕◯◯◯ Very low	Stroke	NRSs, inconsistency
	⊕◯◯◯ Very low	Coronary heart disease	NRSs, imprecision
	⊕◯◯◯ Very low	All-cause mortality	NRSs, inconsistency
	⊕⊕⊕⊕ High	Resting systolic blood pressure	
	⊕⊕⊕⊕ High	Total cholesterol	Not downgraded due to imprecision because follow-up did not cross threshold of relevance of benefit or harm
WHO (sugar intake) [58]*		Effect for reduction in free sugars in adults and children on:	
	⊕⊕⊕◯ Moderate	Bodyweight (follow-up 10 weeks to 8 months)	RoB
	⊕⊕⊕◯ Moderate	Dental caries (follow-up 10 weeks to 8 months)	NRSs, upgraded for large effect size
		Effect of an increase in free sugars intake in adults:	
	⊕⊕⊕◯ Moderate	Bodyweight (follow-up 2 weeks to 6 months)	Potential publication bias
	⊕⊕⊕◯ Moderate	Dental caries (follow-up 1–8 years)	NRSs, upgraded for large effect size
		Effect of a reduction in free sugars intake in children:	
	⊕⊕⊕◯ Moderate	BMI (follow-up 16 to 52 weeks)	Inconsistency
	⊕⊕⊕◯ Moderate	Dental caries (follow-up 1–8 years)	NRSs, upgraded for large effect size
		Effect of an increase in free sugars intake in children:	
	⊕⊕◯◯ Low	Overweight in children	NRSs

*BMI* body mass index, *LDL* low-density lipoprotein, *NRSs* non-randomized studies, *RoB* risk of bias, *SSB* sugar-sweetened beverages

*If more outcomes have been graded, only the seven most important/relevant outcomes per study are presented in this table

**Modified GRADE approach was used. NRSs did not start with “low quality of evidence” but with “high quality of evidence” if NRS study design was the most feasible/optimal one for the intervention

### Survey

To complement the scoping review, a survey was conducted by contacting Ministries of Health or other responsible institutions that play a role in health policymaking in all 53 European countries, as listed by the WHO (Additional file 3) [59]. Contact information was searched through the FAO websites, the WHO websites, and respective websites of national governments. An e-mail was sent in English with a brief description of the project and the question whether the GRADE approach had been used for nutrition and physical activity policymaking in these countries.

## Results

**Fig. 1:**
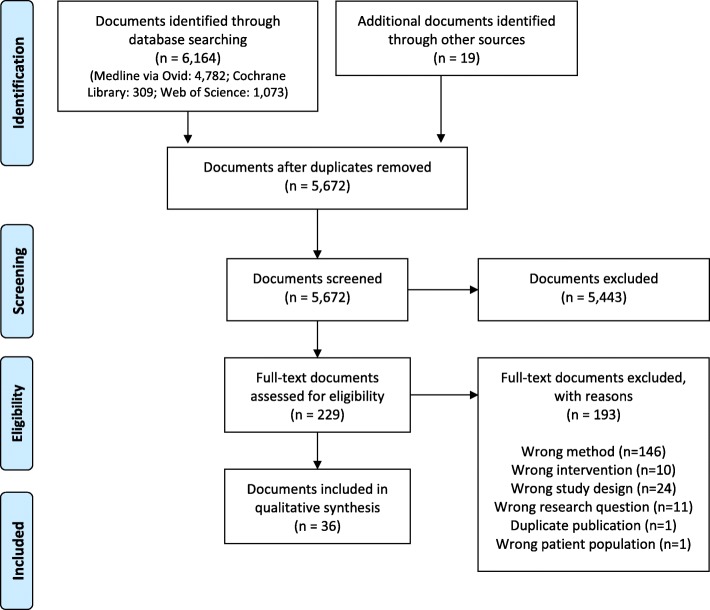
Flow diagram showing study selection process

The database search retrieved 6164 documents (MEDLINE via Ovid, 4782; Cochrane Library, 309; Web of Science, 1073). Another 19 documents were retrieved through further reference checking and from the gray literature. After removal of duplicates (*n* = 511), title and abstract screening of 5672 documents was performed, from which 5443 documents were excluded. The remaining 229 documents were reviewed in full-text screening. The eligibility criteria were met by 36 documents which were included in this scoping review (Fig. 1) [23–58]. The science databases and platforms did not yield any additional documents relevant for this scoping review.

### An overview of the use of GRADE in policymaking and policy evaluation

The characteristics of the included documents are displayed in Table 2. All documents were published in English, and they were either systematic reviews with meta-analyses (*n* = 10; 28%), systematic review protocols (*n* = 8; 22%), systematic reviews without meta-analyses (*n* = 9; 25 %), guidelines (*n* = 6; 17%), an umbrella review (*n* = 1; 3%), or papers on guideline making (*n* = 2; 5%). From these findings, 17 documents (47%) were Cochrane reviews (including five Cochrane review protocols). Four documents were international guidelines by the WHO and one guideline by NICE (physical activity and the environment [45]). Furthermore, two sets of national-level guideline recommendations to guide policymaking were found: the Canadian 24-Hour Movement Guidelines for Children and Youth [52, 53]. Documents were published between 2011 and 2019, and half of those (*n* = 18) were published within the last 3 years. Settings of the studies included communities, schools, or workplaces. The majority of recommendations/interventions addressed the general population through various forms of mass media. Furthermore, the included documents examined various combinations of interventions addressing dietary behavior, physical activity, and sedentary behavior, and also combinations of these with other health-related topics. Most of the studies investigated interventions related to dietary behavior alone (39%), followed by physical activity (17%). There was only one study that assessed interventions aiming to diminish sedentary behavior alone (3%), and all other documents (41%) were based on interventions that targeted various outcomes, focusing on multicomponent interventions that addressed general health and aspects such as alcohol or tobacco consumption.

Many of the documents included did not review implemented policies and focused rather on interventions that have the potential to drive policy in the future. Seven of the 36 documents include studies in which the effects of implemented nutrition and physical activity policies were examined, including nutrition labeling [27, 49], SSB-taxes [35], the WHO Health Promoting Schools framework [39], taxation of fat content in food [40], sodium restriction [43], and taxation of unprocessed sugar or sugar-added foods [48]. Five of these are based on policies issued by governments (see Table 2). Twenty-two of the included documents applied the GRADE approach for grading the certainty of evidence for PEN-related outcomes (for instance, increase of physical activity, change of body-mass index (BMI), and sugar or salt intake) (see Table 3). The remaining 14 documents were either systematic review protocols (*n* = 8, [26, 33, 35, 36, 40, 44, 48, 50]) where GRADE will be used for grading the certainty of evidence, one recommendation to guide policymaking which used GRADE-ADOLOPMENT (GRADE Evidence to Decision frameworks for adoption, adaptation, and de novo development of trustworthy recommendations) (*n* = 1, [47]), documents in which GRADE was used on another development stage of the document (i.e., recommendations where GRADE ratings of included systematic reviews were evaluated, *n* = 2, [52, 53]), or documents in which the barriers against the use of GRADE were stated (*n* = 3, [30, 32, 42]) (see Table 4).

**Table 4:** Facilitators and barriers mentioned by authors

Author	Description
**GRADE not used, barriers mentioned:**	
Matwiejczyk et al. [42]	“Heterogeneity of the reviews and the assessment of insufficient information in three reviews precluded an evaluation of the quality of the research through the use of GRADE. This was compounded by the difficulty for the systematic reviews to apply GRADE or a meta-analysis for the same reason.”
Dyson et al. [30]	“It is acknowledged that much of the evidence from nutrition research is derived from prospective cohort studies rather than RCTs, and applying GRADE downgrades evidence from prospective studies when compared with RCTs; this should be borne in mind when considering the grading allocated to each recommendation.” “A key aspect of current approaches to supporting people with diabetes is to encourage practice that is individualized. It is challenging to rate such recommendations using the GRADE system, particularly in situations where multiple conditions influence health and dietary approaches. In response to this, a deliberate decision was made to report these recommendations as ‘Not Rated’”
Erickson et al. [32]	We planned to use GRADE to evaluate the quality of the evidence used in the model components as well as the accuracy of the modeling procedure; however, these details were not publicly available, and we were unable to assess the quality of the evidence for the recommendations.
**GRADE used, barriers mentioned:**	
Baker et al. [24]	“Given that very few studies had reliable measures of physical activity and sedentary behavior, and much of the data were incomplete, a modified approach was required in which we split the presentation of findings according to the risk of bias. […] As conducting meta-analyses was deemed inappropriate, a summary table has been prepared using narrative analysis of the included studies.”
NICE [45]	It may not be possible, practical or ethical to undertake a randomized controlled trial for some interventions and natural experiments may be the most valid approach. So a modified version of GRADE was agreed by the committee and used. Outcomes from studies for which the natural experiment study design was the most feasible and valid approach started the GRADE process as “high quality.” If a randomized controlled trial was feasible and optimal for answering the study aims but a natural experiment design was used, outcomes started the GRADE process as “low quality.”
**Facilitators of GRADE-ADOLOPMENT approach**	
Okely et al. [47]	The GRADE-ADOLOPMENT approach allows guideline developers to follow a well-accepted and transparent process for developing guidelines (GRADE) in an efficient manner by adapting or adopting an existing evidence-based guideline. This could potentially prevent the need to undertake (or repeat) costly tasks such as conducting full systematic reviews [13]. At the same time, it allows local guideline developers to take into consideration factors that are specific to their local context.

*GRADE* Grading of Recommendations Assessment, Development and Evaluation, *RCT* randomized controlled trial, *GRADE-ADOLOPMENT* GRADE Evidence to Decision frameworks for adoption, adaptation, and de novo development of trustworthy recommendations

Table 3 shows the ratings of the certainty of evidence for all PEN-related outcomes for each study (max. seven ratings per identified document are presented in the table), and the reasons for down- and upgrading. Overall, there were 313 individual outcome ratings (107 GRADE ratings were done for the NICE guideline alone [45]): 34% of very low, 38% of low, 21% of moderate, and 7% of high certainty of evidence. The main reasons for downgrading were high risk of bias, imprecision (e.g., for wide confidence interval, small sample size, and incomplete outcome data), or there was an initial low starting level of a body of evidence from non-randomized study design type (Table 3).

The WHO guidelines were the only documents which, in addition to rating the certainty of evidence for each outcome, also provided the “strength of recommendations” for each recommendation included in the guideline (Additional file 4).

### Facilitators and barriers for the use of GRADE from identified documents

In two documents, an adaption of GRADE was used for primary outcomes [24, 30]. In three documents, the authors mentioned the GRADE approach or presented specific barriers that prevented them from applying GRADE [30, 32, 42], and in one document, the GRADE-ADOLOPMENT approach was used [47] (see Table 4).

Barriers for the use of GRADE for the assessment of the certainty of evidence were mentioned only in six documents. Reasons cited by authors for their decision not to use GRADE included, among others, incomplete, insufficient, or inaccessible data [24, 32, 42]. In another document, it was mentioned that difficulties in the application of the GRADE approach were seen due to the research topic, where RCTs are difficult to conduct, or because of subjective outcome measures such as quality of life [42]. Dyson et al. [30] mentioned some general challenges with the GRADE approach, such as the initial low starting level of a body of evidence from cohort studies and the difficulties of applying GRADE for individualized interventions. Instead, Dyson et al. adopted a grading system very similar to the GRADE system, in which recommendations on the certainty of evidence are provided on a scale from 1 to 4 (strong to very low strength) [30]. The motivation for using this adapted approach is not discussed. In the NICE guideline, a modified version of GRADE was used where NRSs started as “high quality evidence” when the study design was the most feasible one for the examined intervention [45]. Only one document mentioned facilitators for the use of GRADE; however, this was for another GRADE methodology, namely the GRADE-ADOLOPMENT, which was used by Okely et al. for the development of new guidelines [47]. There was no mention of facilitators or barriers for assessing the strengths of recommendations in any of the included documents. Facilitators and barriers are presented in Table 4.

### Complementary survey among European health policymakers

No documents were retrieved through the survey sent to ministries and authorities in 53 European countries. Staff members from eleven countries replied to the request, stating that their national policies were not developed according to the GRADE approach (Additional file 3). Representatives from those countries, namely Austria, Lithuania, Luxemburg, Monaco, the Netherlands, Northern Ireland, and Poland, replied that they do not use GRADE in any way for policymaking. The German Nutrition Society used a newly developed grading system called NutriGrade for their protein guideline [60]. As a response to the survey, Bosnia and Herzegovina showed explicit interest in using GRADE for future policymaking. Iceland and Norway stated that they do not use GRADE for policymaking but in some cases for national clinical guidelines. Monaco replied that they refer to the WHO or French national recommendations. Furthermore, the Nordic Nutrition Recommendations use WHO guidelines, and Norway and Iceland reported that their policies are either based on the Nordic Nutrition Recommendations or they make policies which are based on WHO guidelines directly. No reasons were given whatsoever why GRADE has not been used directly.

## Discussion

### Summary of findings

To the best of our knowledge, this scoping review is the first to examine if and to what extent the GRADE approach has been used in nutrition and physical activity policymaking and policy evaluation in the areas of dietary behavior, physical activity, and sedentary behavior. There were no findings of actual policies or formulation processes from governmental bodies; however, five systematic reviews included evidence from interventions that were based on policies issued by a government or governmental organization (see Table 2). This scoping review found that the GRADE approach has been used for policy evaluations, in the evaluation of the effectiveness of policy-relevant interventions, as well as for guidelines and recommendations intended to guide policymaking, where the evidence for a recommendation or an intervention has to be assessed. These results may contribute to improving the process of evidence-informed policymaking in the areas of dietary behavior, physical activity, and sedentary behavior.

Almost half of the included documents that used the GRADE approach to assess the certainty of evidence are Cochrane Reviews (*n* = 17). The reason for this is that Cochrane adopted the GRADE approach for the evaluation of the certainty of evidence in systematic reviews [61]. The strength of recommendations has only been determined in WHO guidelines, and no barriers to its use were mentioned in these cases. Already in 2003, the WHO selected the GRADE approach as the method of choice to be used in their guideline development [62]. This explains why the guidelines identified in the context of this scoping review are mostly WHO guidelines.

Although guideline and recommendation development are a major part of policymaking, a number of perceived concerns may prevent institutions from adopting available methods. With respect to applying GRADE to policies affecting dietary behavior, physical activity, and sedentary behavior, concerns that are frequently raised include the assessment of risk of bias and the initial low starting level of a body of evidence obtained from NRSs. According to the GRADE approach, the certainty of evidence is initially determined by study design: a body of evidence from RCTs starts with a high certainty, whereas a body of evidence from NRSs, such as prospective observational studies, starts with low certainty due to confounding and selection bias issues associated with NRSs [63, 64]. However, with the advent of tools that use the concept of a target trial as reference point, such as the ROBINS-I (risk of bias in non-randomized studies of interventions), initial certainty of the evidence can also be high for bodies of evidence from NRSs [63, 64]. Nevertheless, as stated above, it is likely that NRSs would be downrated to low certainty because of confounding and selection bias [63].

There has been a long debate regarding what constitutes best evidence in nutrition research and whether it emerges from RCTs in which the effects of a dietary change on disease, intermediate or surrogate markers, or recognized risk markers are evaluated [65]. However, most dietary intervention RCTs are of short duration and do not target patient-relevant outcomes such as morbidity or mortality. RCTs, if well-designed and conducted, provide robust answers to the research questions they address and are considered the ideal methodology for causal inference. NRSs, on the other hand, provide less-robust information regarding causality but are usually considered more applicable for nutrition research. Additionally, all common dietary assessment methods used in nutrition research, such as food consumption records, 24 h recalls, dietary records, dietary history, and food frequency questionnaires suffer from inherent limitations, for example, because the measures are subjective or the results are difficult to reproduce [66].

Apart from the WHO guidelines and the NICE guideline, there were no publications closer to the legislative policymaking process (e.g., policy briefs, policy documents, proposals to inform laws or regulations) that used GRADE. The legislative process underlying policymaking is complex and time-consuming. In addition, there are specific challenges associated with translating empirical findings from the lab into actionable policies in government [66–69]. The absence of documents could be due to various factors: for example, key documents not being publicly available (e.g., internal position papers by political parties), documents being informed by GRADE not explicitly using the term, or the search strategy being specifically tailored to identify those types of policy documents. It could also be due to GRADE not being employed to inform policymakers’ decisions, for example, due to issues in uptake of evidence in the policymaking process [67–69], inadequate knowledge-translation strategies [70–72], or an unawareness about GRADE. Future research needs to address this issue.

### Strengths and limitations

This scoping review has several strengths. Firstly, the inclusion criteria and the search strategy planned for this scoping review were broad, including an extensive search of gray literature such as databases of the WHO or the WCRF in addition to more traditional sources. This access to a wide variety of documents in the research field yields a better overview than previously available. Secondly, we performed a rigorous approach to screening and data extraction, which was performed by two reviewers independently. Thirdly, the survey among national policymakers of the European region brought relevant input and insights into this study by giving an idea about the current use of GRADE within policymaking institutions.

Limitations of this scoping review in our view are as follows. Firstly, developing a suitable search strategy was challenging. Though the search was systematic and as broad as possible, it proved to be difficult to grasp all the facets of the topic. Secondly, only documents were included in which GRADE was applied or reasons were provided for not using GRADE. A different approach with inclusion of documents that could have applied GRADE but did not do so and a research design enabling an in-depth investigation of policymakers’ views (e.g., a mixed methods approach) might have provided further insights into this topic. These approaches would have allowed for a thorough investigation of the extent of evidence use without structured consideration of certainty of evidence and strength of recommendations in policymaking and evaluation. However, this was beyond the scope of this project. Thirdly, a standard definition of the terms “policy” and “health policy” was difficult to determine since various definitions can be found in the literature. Only a few of the included documents (*n* = 7) actually examined real policies. In these cases, the authors did not distinguish between the types of included studies, that is, if an intervention was carried out in an experimental/artificial setting or based on a law/regulation. Lastly, a document was included even when only one of the interventions in the review was considered a policy intervention although the majority of the remaining interventions were not. In this case, ratings for the certainty of evidence with GRADE are likely to be higher than for NRSs in real-life settings as it is much easier to conduct RCTs in experimental settings. 

### Findings from other studies

This is the first scoping review on the use of GRADE in nutrition and physical activity policy evaluation, but other publications have also addressed the complexity of interventions in the public health field, which might prevent researchers and policymakers from applying this approach. Public health and health policy interventions are often thought of as events in systems [73], whereby complex intervention (e.g., characterized by several interacting active components intended to influence multiple behaviors [74]) are implemented in an interactive system (e.g., characterized by changes of the system due to emergent properties, adaptivity, or feedback mechanisms [73, 75]). However, there is specific guidance on how the GRADE approach can be applied in these cases [76, 77].

A study conducted by Rehfuess and Akl in 2013 examined the experience with applying the GRADE approach to public health interventions [77]. In their survey performed in 2013, NICE, which is the national institute that provides guidance and advice to improve health and social care in the UK, stated that the most important reason for not using GRADE when investigating (complex) public health interventions was the fact that most of the evidence originates from NRSs [77]. However, since 2014, NICE included the GRADE approach in their manual for guideline development, also for public health guidelines [78]. Since bodies of NRSs start with low certainty of evidence, it seemed that this would hinder the application of GRADE in such cases [30]. This issue was also brought up in the findings of this scoping review as mentioned above.

Recently, Blake and colleagues showed that from 32 food-based dietary guidelines published since 2010, none of the guidelines used the GRADE approach to evaluate the certainty of evidence or graded the strength of recommendations [79]. However, the authors also acknowledged that methods may not be directly applicable to the types of studies included in food-based dietary guidelines. Of note, GRADE has established working groups focusing on an approach for public health, modeling studies, or environmental exposure studies.

### Implications that follow for the broader research field

It has been pointed out that the use of the GRADE approach in particular in relation to bias assessment is challenging (e.g., users of GRADE may inappropriately double count the risk of confounding and selection bias so the certainty of evidence is downgraded too much) [60, 80]. Further, the usefulness of GRADE to assess the evidence of effectiveness in domains of health policy other than dietary behavior, physical activity, and sedentary behavior needs to be explored. In general, knowledge translation and exchange strategies (i.e., methods of bringing scientific findings to policymakers) have provided mixed results regarding the use or uptake of scientific evidence in the policymaking process [70–72]. One key issue has been the perceived limited relevance, generalizability, and applicability of research findings to a specific setting [81]. Future research should focus on assessing the views of policymakers on the usefulness of GRADE to inform their decisions.

Moreover, beyond the evidence of effectiveness in a given context, public health and health policy decision-makers have to consider numerous additional aspects [82–85], for example, stakeholder interests, ethical concerns, equity/equality considerations, financial implications, or societal and cultural values and norms. These have to be taken into account along with scientific evidence of effectiveness which is often not the most important consideration [68]. In addition, there are individual, group, or systemic factors such as own experiences of policymakers, the political system, institutional mechanisms (structures, processes, or regulations), or contextual, societal factors [69, 86]. The GRADE EtD frameworks might be a suitable approach to address these issues. They might help policymakers to identify, prioritize, and address issues beyond benefit-harm considerations in the healthcare decision-making process in a structured and transparent way such as resource use and cost-effectiveness, equity, acceptability, and feasibility [15, 17]. However, we did not identify any documents which used the EtD frameworks.

## Conclusion

This scoping review was the first attempt to shed some light on how the GRADE approach has been used in nutrition and physical activity policymaking and evaluation. In conclusion, we show that the GRADE approach is able to support the processes of health policymaking (e.g., guidelines) and policy evaluation (e.g., systematic reviews evaluating policy interventions) by facilitating a structured and transparent use of evidence, and that it has already been used in the areas of dietary behavior, physical activity, and sedentary behavior. The aforementioned barriers regarding the use of the GRADE approach have been recognized by the GRADE working group as well, since new tools such as ROBINS-I have been endorsed for the assessment of the certainty of evidence, especially to help decision-makers and policymakers in the area of public health. GRADE is being used as a standard method during guideline development in various institutions, which is an important part in the process of informing policymaking. Nevertheless, our survey shows that GRADE is not being used regularly for actual policymaking. We believe the evaluation of policies should be consistent with standardized methods in order to have the best possible impact on improving health and quality of life, and the GRADE approach could play an important role in this.

## Supplementary information

**Additional file 1.** Preferred Reporting Items for Systematic reviews and Meta-Analyses extension for Scoping Reviews (PRISMA-ScR).

**Additional file 2.** Search in MEDLINE via Ovid, 4 July 2019.

**Additional file 3.** Survey among European health policymakers.

**Additional file 4.** WHO – Strengths and recommendations according to GRADE.

## Data Availability

All data generated or analyzed during this study are included in this published article [and its supplementary information files].

## Abbreviations

BMI: Body mass index
EtD: Evidence to Decision
FAO: Food and Agriculture Organization for the United Nations
GRADE: Grading of Recommendations Assessment, Development and Evaluation
GRADE-ADOLOPMENT: GRADE Evidence to Decision frameworks for adoption, adaptation, and de novo development of trustworthy recommendations
JBI: Joanna Briggs Institute
LDL: Low-density lipoprotein
NCDs: Non-communicable diseases
NICE: National Institute for Health and Care Excellence
NRSs: Non-randomized studies
PEN: Policy Evaluation Network
PRISMA: Preferred Reporting Items for Systematic reviews and Meta-Analyses
RCTs: Randomized controlled trials
ROBINS-I: Risk of bias in non-randomized studies of interventions
SSB: Sugar-sweetened beverages
WCRF: World Cancer Research Fund International
WHO: World Health Organization
