# Supplementary material for: Use of the GRADE approach in health policymaking and evaluation: a scoping review of nutrition and physical activity policies
Source: Implement Sci. 2020 May 24;15:37. doi: 10.1186/s13012-020-00984-2 (PMC7245872; doi:10.1186/s13012-020-00984-2)
Supplement: Supplementary file 2 — Additional file 2. Search in MEDLINE via Ovid, 4 July 2019. [file 13012_2020_984_MOESM2_ESM.pdf]

Additional file 2: Search in Medline via Ovid, 4th of July 2019

1	exp Policy Making/
2	((polic* adj6 making) or (polic* adj6 evaluat*) or (health adj6 polic*) or (polic* adj6 develop*) or (polic* adj6 statement*) or (politic* adj6 strateg*)).ti,ab,kf.
3	(action plan* or policy action* or (promot* adj6 health*) or (polic* adj6 intervent*) or (politic* adj6 intervent*) or (health* adj6 initiativ*) or (intervent* adj6 effic*) or (health* adj6 educati*)).ti,ab,kf.
4	or/1-3
5	exp Nutritional Requirements/
6	exp Nutritional Policy/
7	exp diet/
8	exp Exercise/
9	life style/ or healthy lifestyle/ or healthy aging/ or healthy diet/ or sedentary lifestyle/
10	dedipac.mp.
11	or/5-10
12	4 and 11
13	(grade or grading or recommend* or guideline*).ti,ab,kf.
14	evidence-based practice/ or evidence-based medicine/
15	13 or 14
16	12 and 15
