# Supplementary material for: Use of the GRADE approach in health policymaking and evaluation: a scoping review of nutrition and physical activity policies
Source: Implement Sci. 2020 May 24;15:37. doi: 10.1186/s13012-020-00984-2 (PMC7245872; doi:10.1186/s13012-020-00984-2)
Supplement: Supplementary file 3 — Additional file 3. Survey among European health policymakers. [file 13012_2020_984_MOESM3_ESM.pdf]

Additional file 3: Survey among European health policy makers

**Country**	**Institution**	**Use of GRADE for policy making**
Albania	Ministry of Health	--
Andorra	Ministry of Health, Welfare and Family	
Armenia	Ministry of Health	
Austria	Ministry of Health	Not used
Azerbaijan	Ministry of Health	
Belarus	Ministry of Health	
Belgium	Federal Public Service Health, Food Chain Safety and Environment	--
Bosnia and Herzegovina	Institute of Public Health of Federation of Bosnia and Herzegovina	Not used, but interested in using it
Bulgaria	Ministry of Health	--
Croatia	Ministry of Health	--
Cyprus	Ministry of Health	--
Czech Republic	Ministry of Health	--
Denmark	Ministry of Food, Agriculture and Fisheries	--
Estonia	National Institute for Health Development	--
Finland	National Nutrition Council	--
France	Ministry of Health	--
Georgia	National Centre for Disease Control and Public Health	--
Germany	German Nutrition Society	Nutrigrade
Greece	National and Kapodistrian University of Athens, School of Medicine - WHO Collaborating Center for Food and Nutrition Policies	--
Hungary	National Institute for Food and Nutrition Science	--
Iceland	The Directorate of Health	Not used, but policies are partially based on WHO guidelines
Ireland	Department of Health	--
Israel	Ministry of Health	--
Italy	Research Centre on Food and Nutrition	--
Kazakhstan	Ministry of Healthcare	
Kyrgyzstan	Ministry of Health	
Latvia	Ministry of Health	--
Lithuania	Ministry of Health	Not used
Luxembourg	Ministry of Health	Not used
Malta	The Health Promotion and Disease Prevention Directorate, Parliamentary Secretariat for Health	--
Moldova	Ministry of Health, Labour and Social Protection	
Monaco	Ministry of Health and Social Affairs	Not used, refer to WHO

		and French recommendations
Montenegro	Ministry of Health	
Netherlands	Netherlands Nutrition Centre	Not used
North Macedonia	Institute of Public Health	--
Northern Ireland	Health Development Policy Branch (forwarded by the NHS of the UK)	Not used
Norway	Directorate of Health	Not used for policies, only for guidelines
Poland	National Food and Nutrition Institute	Not used
Portugal	Faculty of Food Sciences and Nutrition, Porto University	--
Romania	National Food and Nutrition Committee, Ministry of Health	--
Russian Federation	Ministry of Health	
San Marino	Authority for health, socio-health and socio-educational services	
Serbia	Ministry of Health	
Slovakia	Public Health Authority	--
Slovenia	National Institute of Public Health	--
Spain	Ministry of Health, Consumption and Social Welfare	--
Sweden	Ministry of Health and Social Affairs	--
Switzerland	Federal Food Safety and Veterinary Office	--
Tajikistan	Ministry of Health and Social Protection	
Turkey	Ministry of Health	--
Turkmenistan	Ministry of Health and Medical Industry	
Ukraine	Ministry of Health	
United Kingdom	National Health Service	--
Uzbekistan	Ministry of Health	
