# Supplementary material for: Use of the GRADE approach in health policymaking and evaluation: a scoping review of nutrition and physical activity policies
Source: Implement Sci. 2020 May 24;15:37. doi: 10.1186/s13012-020-00984-2 (PMC7245872; doi:10.1186/s13012-020-00984-2)
Supplement: Supplementary file 4 — Additional file 4. WHO – Strengths and recommendations according to GRADE. [file 13012_2020_984_MOESM4_ESM.pdf]

Additional file 4: WHO – Strengths and recommendations according to GRADE

WHO Guideline	Recommendations	Strength of recommendation
Potassium intake for adults and children	Increase in potassium intake from food for reduction of blood pressure and risk of cardiovascular disease, stroke and coronary heart disease in adults	Strong
	Potassium intake of at least 90 mmol/day (3510 mg/day) for adults	Conditional
	Increase in potassium intake from food to control3 blood pressure in children	Conditional
Sodium intake for adults and children	Reduction in sodium intake to reduce blood pressure and risk of cardiovascular disease, stroke and coronary heart disease in adults (	Strong
	Reduction to <2 g/day sodium (5 g/day salt) in adults	Strong
	Reduction in sodium intake to control blood pressure in children. The recommended maximum level of intake of 2 g/day sodium in adults should be adjusted downward based on the energy requirements of children relative to those of adults.	Strong
Sugar intake for adults and children	Reduced intake of free sugars throughout the life course	Strong
	Reducing the intake of free sugars to less than 10% of total energy intake2 in both adults and children	Strong
	Further reduction of the intake of free sugars to below 5% of total energy intake	Conditional
Assessing and managing children at primary health-care facilities to prevent overweight and obesity in the context of the double burden of malnutrition	Not providing supplementary foods routinely to moderately wasted infants and children (i.e. with acute undernutrition)	Conditional
	Not providing supplementary foods for treating stunting (chronic undernutrition) among infants and children	Conditional
	Health workers should provide nutrition counselling to caregivers of overweight children aged less than 5 years	Conditional
	Health workers should provide counselling on physical activity to caregivers of overweight children aged less than 5 years	Conditional
	Children aged less than 5 years who are identified as obese should be assessed and an appropriate management plan should be developed. This can be done by a health worker at primary health-care level, if adequately trained, or at a referral clinic or local hospital	Conditional
